# What the electrical impedance can tell about the intrinsic properties of an electrodynamic shaker

**DOI:** 10.1371/journal.pone.0174184

**Published:** 2017-03-22

**Authors:** Bernd Lütkenhöner

**Affiliations:** ENT Clinic, Münster University Hospital, Münster, Germany; Beihang University, CHINA

## Abstract

Small electrodynamic shakers are becoming increasingly popular for diagnostic investigations of the human vestibular system. More specifically, they are used as mechanical stimulators for eliciting a vestibular evoked myogenic potential (VEMP). However, it is largely unknown how shakers perform under typical measurement conditions, which considerably differ from the normal use of a shaker. Here, it is shown how the basic properties of a shaker can be determined without requiring special sensors such as accelerometers or force gauges. In essence, the mechanical parts of the shaker leave a signature in the electrical impedance, and an interpretation of this signature using a simple model allows for drawing conclusions about the properties of the shaker. The theory developed (which is quite general so that it is usable also in other contexts) is applied to experimental data obtained for the minishaker commonly used in VEMP measurements. It is shown that the experimental conditions substantially influence the properties of the shaker. Relevant factors are, in particular, the spatial orientation of the shaker (upright, horizontal or upside-down) and the static force acting on the table of the shaker (which in a real measurement corresponds to the force by which the shaker is pressed against the test person’s head). These results underline the desirability of a proper standardization of VEMP measurements. Direct measurements of displacement and acceleration prove the consistency of the conclusions derived from the electrical impedance.

## Introduction

Small electrodynamic shakers proved to be well suited for diagnostic investigations of the human vestibular system. Shakers used for that purpose are generally fitted with a short plastic rod, which makes it possible to apply mechanical stimuli accurately and safely to specific points on the head [1–3]. Suitable stimuli are, for example, pulses or 500-Hz tone-bursts having a duration of a few milliseconds. The skull vibrations elicited by such stimuli do, of course, not spare the inner ear, which means that they also reach the otolith organs, saccule and utricle. The latter are sensors for linear accelerations, and they play an important role in controlling posture and eye movement. The muscle reflexes elicited by stimulating the otolith organs are widely used for diagnostic testing of the vestibular system. To study these reflexes, electrodes are placed on the skin above (or in close vicinity to) an affected muscle and the so-called vestibular evoked myogenic potential (VEMP) is recorded. Depending on whether a cervical or an ocular muscle is considered, the potential is denoted as cVEMP or oVEMP [4, 5]. The two types of VEMP provide complementary diagnostic information about vestibular disorders [6].

The shaker is commonly hand-held in VEMP investigations. Good contact with the test person’s head is ensured by exerting a force of 10–20 N perpendicular to the surface at the point of contact [2, 3]. This kind of usage significantly differs from the applications for which currently available shakers are designed. Besides, a human head has a mass of the order of 4 kg [7], which is a much heavier “payload” than is normally handled by a small shaker. [The maximum payload considered in the product data sheet for the shaker used here, the Brüel & Kjær mini-shaker type 4810, is 186 g.] Thus, a prediction as to how the shaker performs under the conditions typical for VEMP measurement is problematic. Such concern appears all the more justified as an electrodynamic shaker performs less than ideal even when operated within the certified limits [8–10]. This uncertainty gave rise to the work presented here. Most desirable would, of course, be a model comprising the whole mechanical setup, which consists of the shaker, the hand (and the arm) of the investigator holding the shaker, and the head (as well as the neck) of the test person. With such a model it would be possible, for example, to tailor stimuli that are optimal in some respect. Simulations of that kind could have a significant impact on future VEMP research. However, work towards this goal cannot commence before a solid understanding of the shaker itself is reached. Thus, the question investigated here is how the intrinsic properties of a shaker can be determined from simple measurements under standardized conditions. In the first part of the article, previous modelling approaches [11–15] are adapted to the needs of the situation at hand. Moreover, it is shown how to determine all the model parameters from the electrical impedance of the shaker. In the second part of the article, this theory is applied to measurements taken under a variety of conditions. The consistency of the conclusion derived from the electrical impedance is finally checked by direct measurements of displacement and acceleration.

## Theory

### Time-domain model

**Fig 1:**
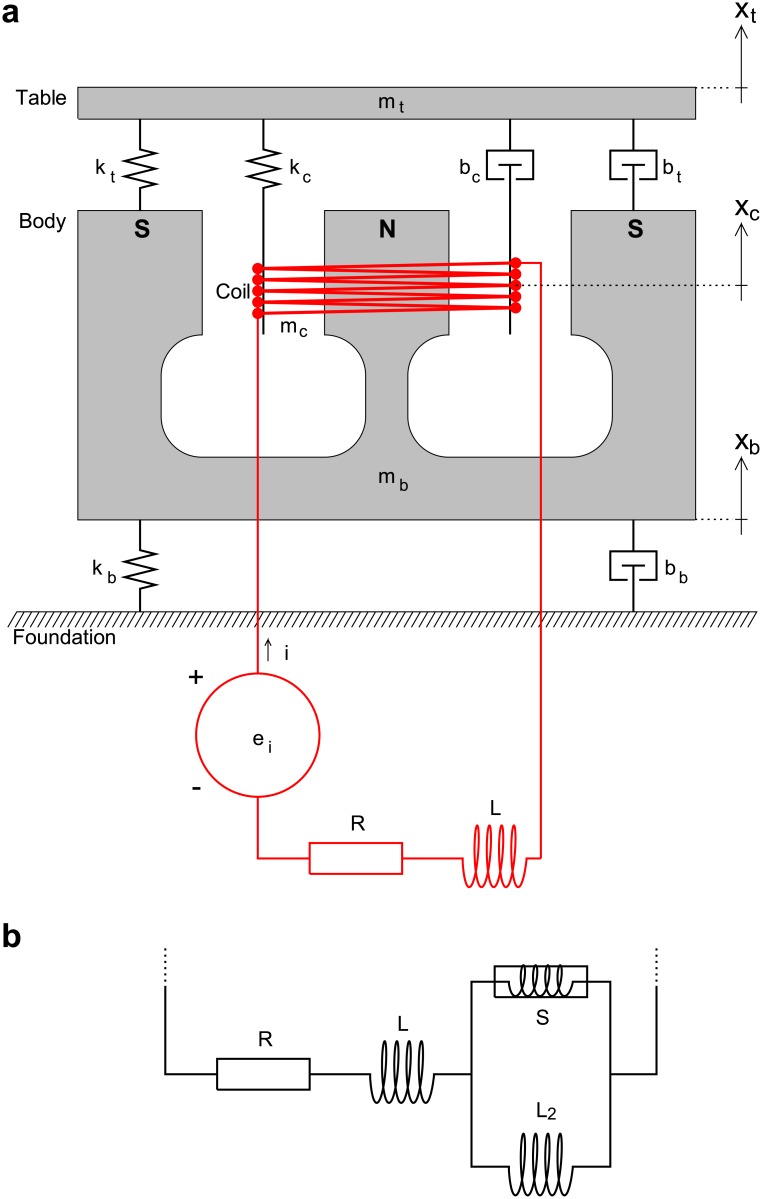
Shaker model. **(a)** The couplings between table and body, coil and table, and body and foundation are each represented by a combination of a spring and a damper; the electrical circuit consists of a voltage source, a resistor, and an inductor (model based on Doebelin [11]). **(b)** Modified electrical circuit that accounts for semi-inductance at higher frequencies. The frequency dependence of the impedance of a semi-inductor is in between that of a resistor (proportional to *ω*^0^, i.e., constant) and that of an inductor (proportional to *ω*^1^). To indicate this fact, the symbol representing the semi-inductor, *S*, is a combination of the symbols for a resistor and an inductor. As suggested by Thorborg and Unruh [16], the semi-inductor is connected in parallel with a second inductor, *L*_2_.

The theory developed here is largely based on the shaker model of Doebelin [11]. Fig 1a shows a schematic drawing of the model considered. From a mechanical point of view, the shaker consists of three structures: table, body, and coil. The associated masses are denoted as *m*_*t*_, *m*_*b*_, and *m*_*c*_. The coupling between table and body is realized by a combination of spring and damper, characterized by the spring constant *k*_*t*_ and the damping coefficient *b*_*t*_. Correspondingly, the coupling between coil and table is characterized by the parameters *k*_*c*_ and *b*_*c*_, and the coupling between body and foundation (this feature represents an extension to the model of Doebelin [11]) by the parameters *k*_*b*_ and *b*_*b*_. The coil is suspended in the magnetic field of a permanent magnet incorporated into the body of the shaker. The poles of the magnet are labeled N (north) and S (south) in the figure. When a current, *i*, is passed through the coil, a force acting in the axial direction is produced, and this force is transmitted to the table attached to the coil. The current is provided by an electrical circuit consisting of a power amplifier with output voltage *e*_*i*_, a resistor *R*, and an inductor *L*. The latter represent the total circuit resistance and inductance, comprising contributions from both the coil and the amplifier [11].

At equilibrium, the weights of table, body, and coil are balanced by restoring forces that are caused by stretching or compressing the springs. Gravitational forces can be ignored when considering deviations from this condition. The displacements of table, body and coil from the respective equilibrium positions are denoted here as *x*_*t*_, *x*_*b*_, and *x*_*c*_. Equations of motion are obtained by applying Newton’s second law, according to which the sum of forces acting on an object equals the mass of that object multiplied by its acceleration. Three types of forces have to be considered. First, the force exerted by a spring is proportional to the distance by which the spring was stretched or compressed (Hooke’s law). Second, a viscous damper exerts a force that is proportional to velocity. Third, a current passed through a coil suspended in a magnetic field exerts a force that is proportional to the current. Applying these laws separately to table, body, and coil, and taking into account that the forces caused by the springs and the dampers oppose movement, yields three second-order differential equations:

$$m_{b}{\ddot{x}}_{b}=-k_{b}x_{b}-k_{t}\left(x_{b}-x_{t}\right)-b_{b}{\dot{x}}_{b}-b_{t}\left({\dot{x}}_{b}-{\dot{x}}_{t}\right)$$ (1)

$$m_{t}{\ddot{x}}_{t}=-k_{t}\left(x_{t}-x_{b}\right)-k_{c}\left(x_{t}-x_{c}\right)-b_{t}\left({\dot{x}}_{t}-{\dot{x}}_{b}\right)-b_{c}\left({\dot{x}}_{t}-{\dot{x}}_{c}\right)$$ (2)

$$m_{c}{\ddot{x}}_{c}=-k_{c}\left(x_{c}-x_{t}\right)-b_{c}\left({\dot{x}}_{c}-{\dot{x}}_{t}\right)+k_{i}i$$ (3)

A fourth differential equation is obtained by considering the voltage in the electrical circuit:

$$e_{i}=iR+L\frac{di}{dt}+k_{i}\left({\dot{x}}_{c}-{\dot{x}}_{b}\right)$$ (4)

The last term on the right-hand side of this equation reflects the fact that a voltage proportional to the velocity of motion is generated when the coil moves relative to the magnet incorporated into the body of the shaker (see, e.g., [12]).

### Driven harmonic motion

If the shaker is driven by an electric potential $e_{i}=E_{i}\exp\left(i\omega t\right)$, the current as well as the displacements of table, body, and coil oscillate at the same frequency, so long as the system is in a steady state. Thus, the solutions to the differential Eqs (1) to (4) have the form $x_{b}=X_{b}\exp\left(i\omega t\right)$, $x_{t}=X_{t}\exp\left(i\omega t\right)$, $x_{c}=X_{c}\exp\left(i\omega t\right)$, and i=Iexp(iωt). All we need to do now is to determine the complex amplitudes *X*_*b*_, *X*_*t*_, *X*_*c*_, and *I*. To begin with, complex amplitude ratios are calculated. For the sake of convenience, they are denoted by the Greek letter *ρ*, with a subscript specifying the complex amplitudes involved. The notations *ρ*_*tb*_ and *ρ*_*ic*_, for example, mean *X*_*t*_/*X*_*b*_, and *k*_*i*_*I*/*X*_*c*_, respectively. Moreover, the notation ${\bar{\rho }}_{tb}$ is introduced for *ρ*_*tb*_ − 1 = (*X*_*t*_ − *X*_*b*_)/*X*_*b*_. Analogous notations apply to other ratios. These notational conventions obviously imply that identities such as *ρ*_*bt*_ = 1/*ρ*_*tb*_ or $-1/{\bar{\rho }}_{bt}=1+1/{\bar{\rho }}_{tb}$ or *ρ*_*bc*_ = *ρ*_*bt*_*ρ*_*tc*_ hold. A combination of such identities yields, e.g.,

$$-\frac{1}{{\bar{\rho }}_{bc}}=1+\frac{1}{{\bar{\rho }}_{cb}}=1+\frac{1}{\rho_{cb}-1}=1+\frac{1}{\left(1+{\bar{\rho }}_{ct}\right)\left(1+{\bar{\rho }}_{tb}\right)-1}.$$ (5)

Because of such identities, only a few fundamental ratios (such as ${\bar{\rho }}_{ct}$ or ${\bar{\rho }}_{tb}$) are required to calculate any other ratio of interest.

Applying these concepts to Eqs (1) and (2) results in

$${\bar{\rho }}_{tb}≔\frac{X_{t}-X_{b}}{X_{b}}=\frac{k_{b}+i\omega b_{b}-\omega^{2}m_{b}}{k_{t}+i\omega b_{t}}$$ (6)

and

$${\bar{\rho }}_{ct}≔\frac{X_{c}-X_{t}}{X_{t}}=\frac{\mu_{0}}{k_{c}+i\omega b_{c}},$$ (7)

with

$$\mu_{0}={\left(\frac{1}{k_{t}+i\omega b_{t}}+\frac{1}{k_{b}+i\omega b_{b}-\omega^{2}m_{b}}\right)}^{-1}-\omega^{2}m_{t}.$$ (8)

Moreover, Eq (3) can be used to relate *X*_*c*_ to the current *I*, yielding

$$\rho_{ic}≔\frac{k_{i}I}{X_{c}}={\left(\frac{1}{\mu_{0}}+\frac{1}{k_{c}+i\omega b_{c}}\right)}^{-1}-\omega^{2}m_{c}.$$ (9)

The relationship between potential *E*_*i*_ and current *I* is usually characterized by means of the ratio *Z* = *E*_*i*_/*I*, called the impedance. From Eq (4) we get

$$Z=R+i\omega L+Z_{m},$$ (10)

where

$$Z_{m}=i\omega k_{i}^{2}/\mu ,$$ (11)

with

$$\mu ≔\frac{k_{i}I}{X_{c}-X_{b}}=\frac{\rho_{ic}}{-{\bar{\rho }}_{bc}},$$ (12)

represents the contribution of the mechanical part of the shaker to the overall electrical impedance (for the calculation of $-1/{\bar{\rho }}_{bc}$ see Eq (5)). Numerical computations can now be done as follows. Given the potential *E*_*i*_, the current is calculated as

$$I=E_{i}/Z.$$ (13)

The other complex amplitudes are then successively calculated as *X*_*c*_ = *k*_*i*_*I*/*ρ*_*ic*_, *X*_*t*_ = *X*_*c*_/*ρ*_*ct*_, and *X*_*b*_ = *X*_*t*_/*ρ*_*tb*_.

### Simplified shaker: Coil and table rigidly attached to one another

The coupling between the coil and the table of the shaker would ideally be rigid [11]. Indeed, shakers are normally designed so that deviations from this ideal condition become noticeable only at high frequencies. Thus, for examinations at lower frequencies it is generally sufficient to consider a simplified shaker, in which coil and table are rigidly attached to one another. This corresponds to the limit *k*_*c*_ → ∞, for which Eq (7) reduces to ${\bar{\rho }}_{ct}=0$, meaning *ρ*_*ct*_ = 1 or *X*_*c*_ = *X*_*t*_. Eq (9) then simplifies to *ρ*_*ic*_ = *μ*_0_ − *ω*^2^*m*_*c*_. Further simplification is achieved by understanding *m*_*t*_ as the combined mass of coil and table. With such a redefinition of *m*_*t*_, Eq (9) can be replaced by *ρ*_*ic*_ = *μ*_0_. The displacement of the table can then be calculated more directly as

$$X_{t}=\frac{k_{i}I}{\mu_{0}},$$ (14)

and Eq (12) can be rewritten as

$$\mu =\frac{k_{i}I}{X_{t}-X_{b}}=\frac{\mu_{0}}{1-\rho_{bt}}.$$ (15)

The body of the shaker typically shows much smaller displacements than the table so that the approximation *ρ*_*bt*_ ≈ 0 can be used. This eventually yields *μ* ≈ *μ*_0_.

### Resonance frequencies

A first guess of the resonance frequencies to be expected (in this theoretical part of the article, the term ‘frequency’ always means ‘angular frequency’) is obtained by decomposing the shaker into three mass-spring systems, each one representing one of the mechanical components: table, body, and coil. The resonance frequency of an undamped mass-spring system is

$$\omega_{0}\left(k,m\right)=\sqrt{k/m},$$ (16)

where *k* and *m* denote the spring constant and the mass of the system [17, 18]. Thus, the shaker is expected to show resonance when it is driven at frequencies around *ω*_*t*_ = *ω*_0_(*k*_*t*_, *m*_*t*_), *ω*_*b*_ = *ω*_0_(*k*_*b*_, *m*_*b*_), and *ω*_*c*_ = *ω*_0_(*k*_*c*_, *m*_*c*_), respectively.

These estimates are, of course, rough approximations at best, because interactions between body, table, and coil are ignored. The purpose of the following considerations is to get a more realistic idea. For the sake of simplicity, the analysis is confined to the case that coil and table are rigidly attached to one another. This means that Eq (14) is applicable. Provided that the current, *I*, is roughly constant in the frequency range of interest (as is the case if the impedance is dominated by the resistance *R*), Eq (14) suggests that the resonance frequencies of the shaker can be determined by calculating the minima of |*μ*_0_|. To further simplify the calculations, mechanical damping is considered negligible, i.e., *b*_*b*_ = *b*_*t*_ = 0. Eq (8) then becomes

$$\mu_{0}\left(\omega \right)=m_{t}\;\left({\left(\frac{1}{\omega_{t}^{2}}+\frac{m_{t}/m_{b}}{\omega_{b}^{2}-\omega^{2}}\right)}^{-1}-\omega^{2}\right).$$ (17)

The resonance frequencies are now found by determining the roots of *μ*_0_(*ω*). This task is facilitated by introducing the auxiliary quantity

$$\Omega =\frac{\omega_{b}}{\omega_{t}}+\frac{\omega_{t}}{\omega_{b}}\;\left(1+\frac{m_{t}}{m_{b}}\right)$$ (18)

and rewriting Eq (17) as

$$\mu_{0}\left(\omega \right)=\frac{m_{t}\omega_{b}^{2}\omega_{t}^{2}}{\omega_{b}^{2}+\omega_{t}^{2}\;m_{t}/m_{b}-\omega^{2}}\;\left[{\left(\frac{\omega^{2}}{\omega_{b}\omega_{t}}\right)}^{2}-\frac{\omega^{2}}{\omega_{b}\omega_{t}}\Omega +1\right].$$ (19)

As to the roots of *μ*_0_(*ω*), only the term in square brackets is relevant, which means that a quadratic equation in *ω*^2^ has to be solved. The two solutions are

$$\omega_{\pm }^{2}=\frac{\omega_{b}\omega_{t}}{2}\;\left(\Omega \pm \sqrt{\Omega^{2}-4}\right).$$ (20)

The inequalities

$$\Omega^{2}-4>\;{\left(\frac{\omega_{b}}{\omega_{t}}+\frac{\omega_{t}}{\omega_{b}}\right)}^{2}-4=\;{\left(\frac{\omega_{b}}{\omega_{t}}-\frac{\omega_{t}}{\omega_{b}}\right)}^{2}\geq 0$$ (21)

and $\sqrt{\Omega^{2}-4}<\Omega$ ensure that both $\omega_{+}^{2}$ and $\omega_{-}^{2}$ are greater than zero, for all conceivable parameter constellations.

Supposing that *ω*_*t*_ and *ω*_*b*_ differ by at least an order of magnitude, Eq (20) can be replaced by simple approximations. The situation that the body of the shaker is more or less rigidly attached to the foundation corresponds to the assumption *ω*_*t*_/*ω*_*b*_ ≪ 1. A series expansion then leads to the approximations $\omega_{+}^{2}\approx \omega_{b}^{2}$ and $\omega_{-}^{2}\approx \omega_{t}^{2}$. Only the latter approximation matters here, because the assumption *ω*_*b*_ ≫ *ω*_*t*_ normally implies that *ω*_*b*_ is outside the frequency range of interest. With regard to a hand-held shaker, the reverse condition is of interest: Assigning a low stiffness to the coupling between the body of the shaker and the “foundation” (the latter being represented by the investigator’s body) is equivalent to assuming *ω*_*b*_/*ω*_*t*_ ≪ 1. A series expansion for this condition leads to the approximations

$$\omega_{+}^{2}\approx \omega_{t}^{2}\cdot \;\left(1+m_{t}/m_{b}\right)=k_{t}\;\left(1/m_{b}+1/m_{t}\right)$$ (22)

and

$$\omega_{-}^{2}\approx \omega_{b}^{2}\;/\;\left(1+m_{t}/m_{b}\right)=k_{b}/\left(m_{b}+m_{t}\right).$$ (23)

According to these approximations, the upper resonance frequency, *ω*_+_, is higher than the resonance frequency of the isolated table, whereas the lower resonance frequency, *ω*_−_, is lower than the resonance frequency of the isolated body. Note that, in these approximations, the upper resonance frequency does not depend on *k*_*b*_, whereas the lower resonance frequency does not depend on *k*_*t*_. The assumption *ω*_*b*_ ≪ *ω*_*t*_ normally implies that *ω*_−_ is outside the frequency range of interest.

### Estimation of the shaker parameters from measurements of the electrical impedance

From Eq (10) it can be concluded that by measuring the frequency dependence of the electrical impedance it is possible to determine not only the electrical, but also the mechanical properties of the shaker. Indeed, regarding the simplicity of the first two terms on the right-hand side of the equation, *Z* represents, in essence, a fingerprint of the mechanical part of the shaker, which determines the third term. A complicating issue is that the third term has more parameters than can be estimated from the data of a single experiment. To get around this problem, a different parametrization is needed.

The intrinsic properties of the shaker are preferably studied under conditions where confounding factors are avoided as far as possible. The following analysis is therefore confined to the case that the shaker is rigidly attached to the foundation. In the model, this situation corresponds to the limit *k*_*b*_ → ∞. Eq (8) reduces, under such circumstances, to $\mu_{0}=k_{t}+i\omega b_{t}-\omega^{2}m_{t}$. Moreover, in the case of the simplified shaker model, *μ* can be replaced by *μ*_0_ in Eq (11). If *ω*_*t*_ is the resonance frequency of the isolated table, as defined above, and

$$\zeta_{t}=\frac{b_{t}}{2\sqrt{m_{t}k_{t}}}$$ (24)

is the corresponding damping ratio [17, 19], the formula for *μ*_0_ can be rewritten as

$$\mu_{0}=m_{t}\;\left(\omega_{t}^{2}-\omega^{2}+2i\omega \omega_{t}\zeta_{t}\right).$$ (25)

Thus, all in all, Eq (11) can be replaced by

$$Z_{m}=\frac{{\tilde{k}}_{i}^{2}}{2\omega_{t}\zeta_{t}+i\left(\omega^{2}-\omega_{t}^{2}\right)/\omega },$$ (26)

with

$${\tilde{k}}_{i}^{2}=k_{i}^{2}/m_{t}.$$ (27)

This reparameterization reduces the number of parameters by one, and model fitting is possible irrespective of whether or not the mass of the table, *m*_*t*_, is known. If so, the original parameters (*k*_*t*_, *b*_*t*_, *k*_*i*_) are easily calculated using Eqs (16), (24) and (27). If not, it can be exploited that attaching a payload to the table changes the resonance frequency. Such an approach requires that *m*_*t*_ is understood as the combined mass of table and payload, i.e.,

$$m_{t}=m_{T}+m_{P},$$ (28)

where *m*_*T*_ is the mass of the table alone and *m*_*P*_ is the mass of the payload. Applying Eq (16) we then get

$$\omega_{t}^{-2}=\left(m_{T}+m_{P}\right)/k_{t}.$$ (29)

Provided that data is available for at least two different values of *m*_*P*_, the linear dependence of $\omega_{t}^{-2}$ on *m*_*P*_ offers an easy way to determine both *m*_*T*_ and *k*_*t*_ (for details see the experimental section below).

### Closer examination of the table resonance

The quantity *Z*_*m*_ as specified in Eq (26) is ideally suited for studying the table resonance. Multiplying *Z*_*m*_ and its complex conjugate yields

$$|Z_{m}{|}^{2}=\frac{{\tilde{k}}_{i}^{4}}{4\omega_{t}^{2}\zeta_{t}^{2}+{\left(\omega^{2}-\omega_{t}^{2}\right)}^{2}/\omega^{2}}.$$ (30)

It is evident from this formula that |*Z*_*m*_| reaches its maximum when the second term in the denominator vanishes: for *ω* = *ω*_*t*_. The value of this maximum is

$$\max\;\left(|Z_{m}|\right)=Z_{m}\left(\omega_{t}\right)=k_{i}^{2}/b_{t}.$$ (31)

Provided that the electrical impedance *Z* is dominated by the electrical resistance *R* (so that the inductance term, iωL, is negligible for the frequency range of interest), the maximum of |*Z*_*m*_| coincides with that of the table velocity $|{\dot{X}}_{t}|$. In order to show this, we first substitute *E*_*i*_/*Z* for *I* in Eq (14). The approximation *Z* ≈ *R* + *Z*_*m*_ combined with Eqs (25) to (27) then yields

$$\frac{X_{t}}{E_{i}}\approx \frac{c/m_{t}}{\omega_{t}^{2}-\omega^{2}+i\omega \left[Rc^{2}/m_{t}+2\omega_{t}\zeta_{t}\right]},$$ (32)

where

$$c=k_{i}/R$$ (33)

is a constant having the units N/V. For the table velocity, ${\dot{X}}_{t}=i\omega X_{t}$, this means:

$$\frac{{\dot{X}}_{t}}{E_{i}}\approx \frac{c/m_{t}}{Rc^{2}/m_{t}+2\omega_{t}\zeta_{t}+i\left(\omega^{2}-\omega_{t}^{2}\right)/\omega }.$$ (34)

The right-hand side of this equation has essentially the same structure as the right-hand side of Eq (26). Hence, $|{\dot{X}}_{t}|$ reaches its maximum at the same frequency as *Z*_*m*_: at *ω* = *ω*_*t*_. In both cases the maximum is caused by the real part, whereas the corresponding imaginary part is zero. The maximum of the table displacement, $|X_{t}\left|=\right|{\dot{X}}_{t}|/\omega$ typically occurs at a frequency that is somewhat lower than *ω*_*t*_, whereas the maximum of the table acceleration, $|{\ddot{X}}_{t}\left|=\omega \right|{\dot{X}}_{t}|$, typically occurs at a somewhat higher frequency.

Eq (30) can be rewritten as

$$|Z_{m}|=\frac{{\tilde{k}}_{i}^{2}}{2\omega_{t}\zeta_{t}}\;{\left(1+\;{\left(\frac{\xi }{\zeta_{t}}\right)}^{2}\right)}^{-1/2},$$ (35)

where

$$\xi ≔\frac{\omega^{2}-\omega_{t}^{2}}{2\omega \omega_{t}}=\frac{1}{2}\;\left(\frac{\omega }{\omega_{t}}-\frac{\omega_{t}}{\omega }\right)$$ (36)

is basically a normalized frequency difference. [With Δ*ω* = *ω* − *ω*_*t*_ we get 2*ξ* = (1 + Δ*ω*/*ω*_*t*_) − (1 + Δ*ω*/*ω*_*t*_)^−1^. For Δ*ω* ≪ *ω*_*t*_, the latter term can be approximated as 1 − Δ*ω*/*ω*_*t*_. Thus, we finally get *ξ* ≈ Δ*ω*/*ω*_*t*_.] The full width at half maximum (FWHM) of |*Z*_*m*_|, measured on the *ξ* scale rather than the *ω* scale, is easily calculated as

$${\text{FWHM}}_{\xi }=2\sqrt{3}\zeta_{t}.$$ (37)

This equation offers a simple possibility for numerically determining the parameter *ζ*_*t*_ from given data.

### Simple approximations for the acceleration of the table

Users of a shaker are generally more interested in the acceleration achieved by the table rather than the impedance of the electrical circuit. It is noteworthy therefore that, for frequencies being much lower or higher than the resonance frequency, a rough estimate of the acceleration can be obtained using very simple approximations, provided that Eq (32) is applicable. The approximation for low frequencies (*ω* → 0) is

$${\ddot{X}}_{t}/E_{i}\approx -\omega^{2}c/k_{t},$$ (38)

and the approximation for high frequencies (*ω* → ∞) is

$${\ddot{X}}_{t}/E_{i}\approx c/m_{t}.$$ (39)

The latter approximation offers an especially easy way to determine the parameter *c* by accelerometry.

### A complicating issue: semi-inductance

In the experimental section of this article it will be shown that the assumption of a resistor and an inductor in series (as in Fig 1a) is not sufficient to explain the electrical impedance measured at higher frequencies. Similar conclusions were reached in studies of electrodynamic loudspeakers, which bear some resemblance to the shaker considered here [14]. Vanderkooy [20] explained the peculiarities observed at higher frequencies by eddy currents in the iron pole structure of the speaker. Several authors tried to mimic the effect by means of simple analogue circuits [16, 21–24]. Here, a solution developed by Thorborg and Unruh [16] is employed. Their model makes use of a so-called semi-inductor. While in the case of an inductor the impedance is proportional to frequency and the phase of the current lags the phase of the voltage by 90 degrees, the impedance of a semi-inductor is proportional to the square root of frequency, and the phase lag is 45 degrees [25]. To implement the model of Thorborg and Unruh [16], the electrical circuit is modified as indicated in Fig 1b. Eq (10) then has to be replaced by

$$Z=R+i\omega L+Z_{\text{semi}}+Z_{m},$$ (40)

where the term

$$Z_{\text{semi}}=\;{\left(\frac{1}{i\omega L_{2}}+\frac{1}{\sqrt{i\omega }S}\right)}^{-1}$$ (41)

allows for modeling semi-inductance. While *L*_2_ is measured in units of henry, *S* is measured in units of semihenry [25]. For fitting the model to experimental data it is convenient to rewrite Eq (41) as

$$Z_{\text{semi}}=\frac{i\omega L_{2}}{1+\sqrt{i\omega /\omega_{semi}}},$$ (42)

with $\omega_{semi}=S^{2}/L_{2}^{2}$. In the latter formula, *Z*_semi_ is linearly dependent on *L*_2_, whereas the other parameter, *ω*_*semi*_, controls the transition from $Z_{\text{semi}}\approx i\omega L_{2}$ (valid for *ω* → 0) to $Z_{\text{semi}}\approx {\left(i\omega \right)}^{1/2}S$ (valid for *ω* → ∞).

## Methods

### Measurement hardware

The shaker studied was a Brüel & Kjær mini-shaker Type 4810. As specified by the manufacturer, the dynamic weight of the moving system is 18 g, whereas the total weight is 1.1 kg. The shaker was driven by a Brüel & Kjær power amplifier Type 2718. This amplifier has a flat frequency response from 10 Hz to 20 kHz (±0.5 dB). The input to the power amplifier was provided by a TDT RP2.1 real-time processor (Tucker-Davis Technologies, Alachua, FL), which has 24-bit digital-to-analog (D/A) converters. The power amplifier allows for monitoring both the output voltage and the current in the electrical circuit. These two signals were fed to the 24-bit analog-to-digital (A/D) converters of the real-time processor. The real-time processor communicated with a PC via USB.

### Measurement software

The real-time processor executed low-level assembly code that was generated using the graphical design interface RPvdsEx (Tucker-Davis Technologies, Alachua, FL). In essence, this code served to send out a discrete-time signal to a D/A converter while simultaneously acquiring data from the A/D converters. The real-time processor was controlled by custom Matlab software running on the PC. The Matlab software not only generated the discrete-time signal for the D/A converter (sent to the real-time processor before starting the measurement), but also saved the output streams of the A/D converters to a hard disk drive.

The signal sent to the D/A converter was a tone-burst. Both the rise time and the fall time corresponded to one cycle of the tone. Tone-bursts with a frequency greater than 80 Hz had a plateau duration of 100 ms; for lower frequencies the plateau duration corresponded to eight cycles of the tone (this means, for example, that a 1-Hz tone-burst had a plateau duration of 8 seconds). To determine the frequency dependence of the electrical impedance, the frequency of the tone-burst was generally varied between 1 Hz and 5 kHz, using 48 discrete frequencies per octave (all in all yielding 590 discrete frequencies; overall measurement time: about 30 minutes). If not stated otherwise, the tonebursts were amplified such that the maximum input to the shaker was 1 V. This amplification level will be referred to as 0 dB. The digitization rate was about 48.8 kHz for the investigation of frequencies above 500 Hz and about 9.8 kHz for the investigation of lower frequencies.

### Mechanical setup

In the main experiments, the shaker was firmly mounted on a solid foundation having a mass of about 10 kg (two 5-kg counterweights of an astronomical telescope mount were attached together). Measurements were done for three different orientations of the shaker: upright, upside-down, and horizontal. For the conditions ‘upright’ and ‘horizontal’, the foundation was placed on the floor of the laboratory. For the condition ‘upside-down’, the foundation was supported by two heavy pedestals. The latter were arranged so that the space between them allowed for mounting the shaker underneath the foundation. The moving mass was systematically varied by screwing different objects to the table of the shaker. The most heavy object was a solid brass cylinder with a mass of 1073 g, which is roughly the total mass of the shaker itself. Two other brass cylinders had masses of 487 g and 243 g, respectively. The three brass cylinders, which were specially manufactured for this project, will be referred to as the 1100-g, the 500-g, and the 250-g payload. In order to become more flexible as to the choice of the weights, also a small vice was manufactured (mass 93.1 g). The vice made it possible to attach arbitrary small objects to the table (mainly washers of different sizes were used). Masses smaller than that of the vice were realized using various combinations of screw-nuts and washers. The masses attached to the table were determined using a letter scale with a digital display.

Supplementary experiments were done with the shaker hold in the hand of an investigator (the author himself). To keep the overall measuring time within acceptable limits, only a few exemplary conditions were considered. Moreover, the measuring time per condition was substantially reduced by limiting the investigation to the frequency range between 10 Hz and 1000 Hz (319 frequencies; overall measuring time per condition: about 5 minutes).

### Data analysis

After completing the measurements, impedances were calculated from the stored data, i.e., the simultaneous recordings of the amplifier output voltage and the current in the electrical circuit. The calculations (successively done for all frequencies and all experimental conditions) were based on the assumption that the shaker operated under steady-state conditions during the second half of a tone-burst presentation. In a first step, a complex amplitude was estimated separately for the voltage and the current. This was done by means of a least-squares fit in the time domain, confined to the time range that corresponds to the second half of the plateau. [It is straightforward to find an analytical solution for this linear optimization problem. Great care was used to correct for the hardware-specific delays associated with D/A and A/D conversion.] In the second step, the impedance was calculated by dividing the complex amplitude estimated for the voltage by the complex amplitude estimated for the current.

In a subsequent step, which was performed separately for each experimental condition, the frequency dependence of the measured impedance was interpreted by fitting a model to the data. This was done using the Matlab routine fminsearch, which employs the simplex search method of Lagarias et al. [26]. The function minimized by this routine corresponded to the sum of the squared differences between the measured impedances and the impedances predicted by the model (real and imaginary part were handled as two separate data values). The model impedance generally corresponded to Eq (10), with *Z*_*m*_ as specified in Eq (26). Thus, all in all, five parameters had to be estimated: *R*, *L*, ${\tilde{k}}_{i}^{2}$, *ω*_*t*_, and *ζ*_*t*_. The routine fminsearch explicitly handled only the optimization of the latter two parameters, because for almost each choice of *ω*_*t*_ and *ζ*_*t*_, optimal values for *R*, *L*, and ${\tilde{k}}_{i}^{2}$ are easily determined by solving a linear optimization problem (Matlab routine regress was used for that purpose). An initial value for *ω*_*t*_ was obtained by numerically determining the root of the imaginary part of the measured impedance. Using this rough estimate for *ω*_*t*_, the *ω* scale was transformed into the *ξ* scale (cf. Eq (36)), and FWHM_*ξ*_ was estimated from the measured impedances. An initial value for *ζ*_*t*_ was finally obtained from Eq (37).

If the theoretical impedance was given by Eq (40), with *Z*_semi_ as specified in Eq (42), fminsearch also had to determine the parameter *ω*_*semi*_. An initial value for this parameter was obtained by trial and error. The parameter *L*_2_ was estimated by linear optimization, simultaneously with *R*, *L*, and ${\tilde{k}}_{i}^{2}$.

### Supplementary measurements of acceleration and static displacement

To check the consistency of the acceleration predicted from the measured electrical impedance, acceleration measurements were carried out by connecting a Brüel & Kjær accelerometer (type 4535-B-001, operated through a signal conditioner type 1704-A-002) rather than the voltage monitor of the power amplifier to the A/D converter module. Apart from that, the procedure was analogous to the measurement of the electrical impedance.

Another consistency check was done by measuring the static displacement caused by attaching a weight to the table of the shaker. The length measurements were done with a digital calliper having an error limit of 0.03 mm (MarCal 16 EWR, Mahr GmbH, Esslingen, Germany). To reduce the measurement error caused by manually placing the tips of the calliper on specific reference points of the shaker, the results of 10 independent measurements were averaged.

## Results

### Electrical impedance for an exemplary condition

**Fig 2:**
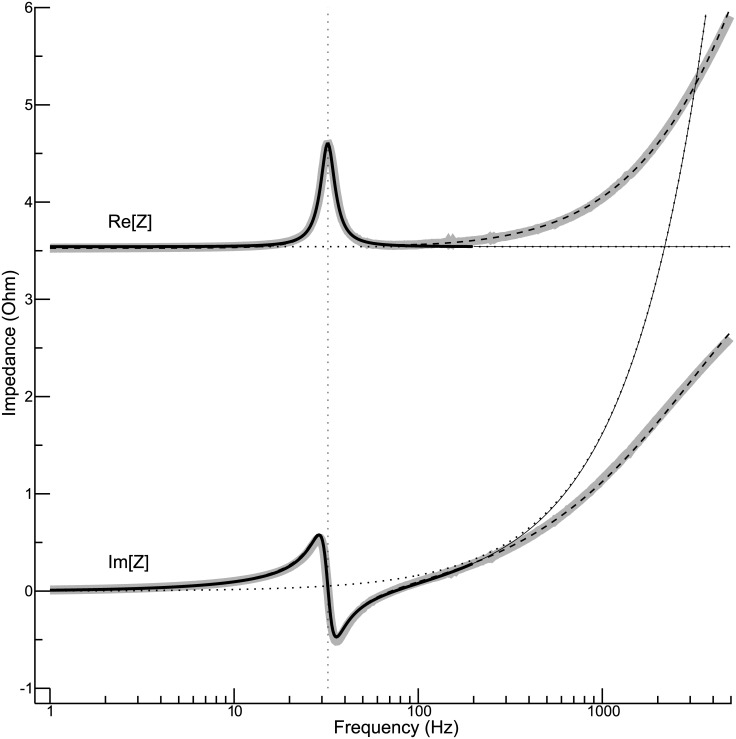
Exemplary impedance function. The real and the imaginary part of the impedance are shown as functions of frequency. The thick grey curves in the background represent the measured data. The other curves represent various model fits. The shaker was in an upright position, and the 250-g payload was used.

Fig 2 shows the frequency dependence of the electrical impedance for an exemplary condition. The shaker was in an upright position, and the 250-g payload was attached to its table. Real and imaginary part are considered separately. The thick grey curves represent the measured data, whereas the other curves show different model fits. The solid black curves were obtained for the standard model represented by Eq (10), with *Z*_*m*_ as defined in Eq (26). The frequency range accounted for by the fit algorithm was 1 to 200 Hz (indicated by an increased line thickness). The model reproduces the data quite well in this frequency range. Most conspicuous is the concurrence of a pronounced peak of the real part and a root of the imaginary part. This feature can be explained by a resonance of the table (more precisely: table with attached payload). The estimated resonance frequency is 32.25 Hz, and the estimated damping ratio is *ζ*_*t*_ = 0.109. The other parameters provided by the fit algorithm are *R* = 3.54 Ω, *L* = 258.6 *μ*H, and ${\tilde{k}}_{i}^{2}=47.02\;{\left(\text{N}/\text{A}\right)}^{2}/\text{kg}$. Although the exact mass of the table is unknown at this point, Eq (16) allows us to derive at least a first estimate of the shaker parameter *k*_*t*_, because the attached payload was an order of magnitude heavier than the table itself. Assuming that table plus attached payload have a mass of about 0.26 kg, a value of 10.7 N/mm is estimated for *k*_*t*_. The Eqs (24) and (27) then provide the values 11.5 N/(m/s) and 3.50 N/A for the parameters *b*_*t*_ and *k*_*i*_, respectively.

The contribution of the mechanical part of the shaker to the electrical impedance is negligible for *ω* ≫ *ω*_*t*_ [from Eq (26) it follows that |*Z*_*m*_| → 0 for *ω* → ∞]. Thus, for high frequencies one would expect that the real part of the impedance is basically equal to *R* and that the imaginary part is basically equal to *ωL*. These expectation are represented by dotted curves in Fig 2 (mostly coinciding with the solid curves). But the measured data do not confirm this expectation. Instead of approaching a constant value, the real part of the impedance begins to increase at about 100 Hz, and this increase accelerates with increasing frequency. The imaginary part of the impedance shows a similar increase, which, however, falls short of the expected increase. These discrepancies force us to conclude that a crucial aspect is missing in the standard model. The problem can be largely solved by using Eq (40) instead of Eq (10), which means that the electrical circuit is assumed to comprise a semi-inductive component. The dashed curves in Fig 2 show that, with this extension, the model fits the data fairly well in the frequency range considered. [The values estimated for *L*, *L*_2_, and *ω*_*semi*_ are -61 *μ*H, 414 *μ*H, and 1.77 kHz, respectively. The other parameter values roughly correspond to the standard model. Taken by itself, the negative value of the inductance *L* makes no sense. But the apparent inconsistency dissolves once the terms iωL and *Z*_semi_ in Eq (40) are combined: The overall inductance in the low-frequency limit is *L* + *L*_2_ ≈ 353 *μ*H.] Nevertheless, considering the fact that this study focuses on the mechanical properties of the shaker, which affect the electrical impedance mainly at lower frequencies, the assumption of a semi-inductor represents a complication that is better avoided. All the following analyses are therefore confined to frequencies below 200 Hz.

### Impedance curves for different payloads

**Fig 3:**
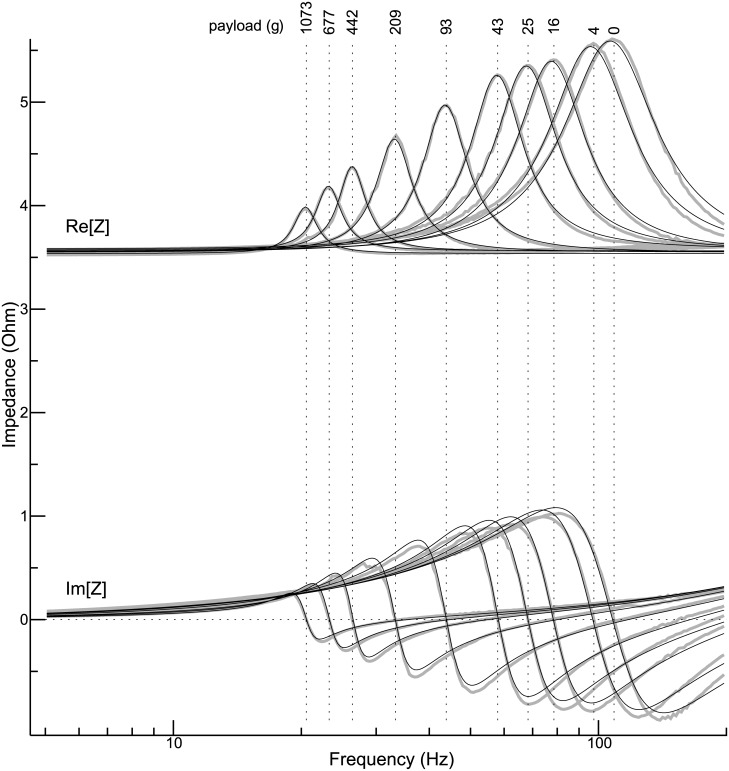
Family of impedance curves. The curves are displayed in basically the same way as the exemplary curves in Fig 2. The payload was systematically varied between 0 and 1.073 kg.

Fig 3 illustrates how varying the payload attached to the table alters the impedance curves. The shaker was again in an upright position. As expected (cf. Eq (16)), the resonance frequency is the lower, the higher the payload is: Without payload, the resonance frequency is 106.5 Hz, whereas with the highest payload (1.073 kg) a resonance frequency of 20.45 Hz is observed. Another striking finding is that the contribution of the mechanical part of the shaker to the overall electrical impedance decreases with increasing payload. Eq (31) suggests that this effect is caused by an increase of the damping coefficient *b*_*t*_ (assuming that the parameter *k*_*i*_ is basically constant). Considering the simplicity of the model, the fit between data and model is satisfying.

### Estimation of the mass of the table

**Fig 4:**
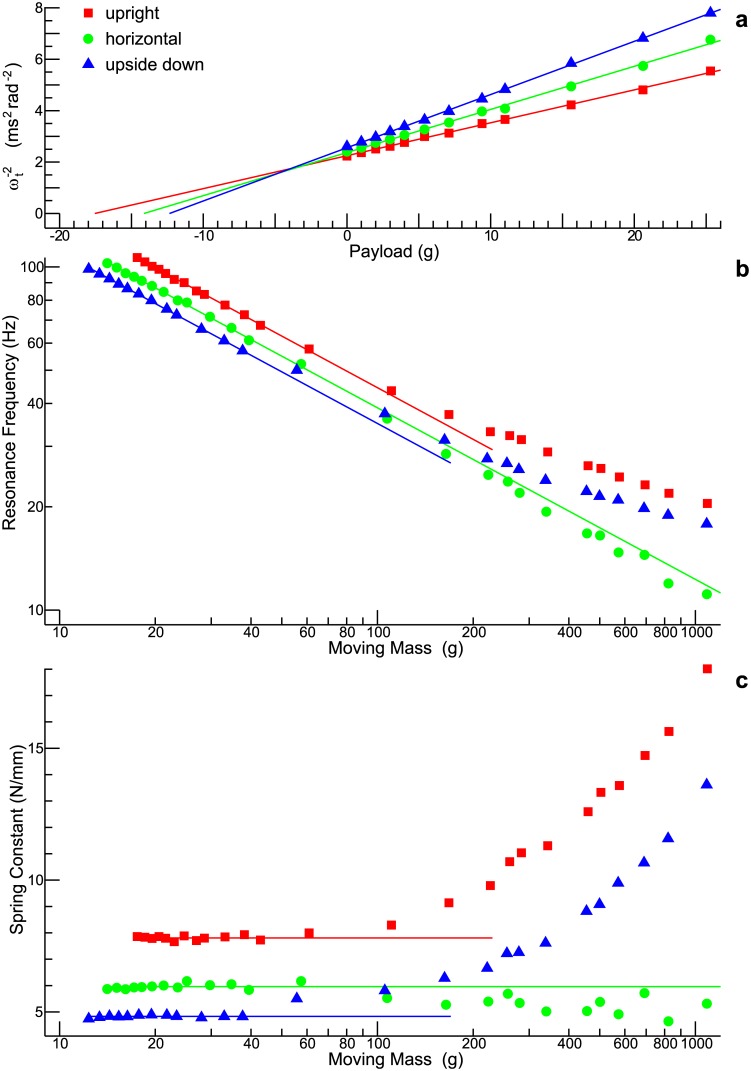
Parameter estimation. **(a)** Estimation of the mass of the table, *m*_*T*_. The data obtained for small payloads confirms the theoretical prediction that the squared reciprocal of the resonance frequency, $\omega_{t}^{-2}$, is a linear function of the payload, with an intercept corresponding to −*m*_*T*_. What was unanticipated is the substantial discrepancy between the results for the three spatial orientations of the shaker (upright, horizontal, and upside-down). **(b)** Resonance frequency as a function of the moving mass. **(c)** Spring constant as a function of the moving mass.

In the theoretical part of this work it is generally assumed that the combined mass of table and payload is known. Thus, in order to apply the equations derived, first of all the mass of the table itself, *m*_*T*_, has to be estimated. Eq (29) is particularly suited for this purpose, because this equation predicts a linear relationship between the payload, *m*_*P*_, and the squared reciprocal of the resonance frequency, $\omega_{t}^{-2}$. Fig 4a shows experimental data for three different spatial orientations of the shaker: upright, horizontal, and upside-down. The maximum payload in these experiments was 25.3 g (slightly larger than the expected mass of the table). The spatial orientation of the shaker turned out to be a decisive factor so that the analysis had to be done separately for each condition. All in all, the data confirm the expectation that $\omega_{t}^{-2}$ (represented by the ordinate) is a linear function of the payload (represented by the abscissa). By fitting a straight line to the data (Matlab routine polyfit) it was possible to determine the parameters *m*_*T*_ and *k*_*t*_. The estimates obtained for *m*_*T*_ are 17.5 g (upright), 14.0 g (horizonzal), and 12.2 g (upside-down). The corresponding estimates for *k*_*t*_ are 7.81 N/mm, 5.91 N/mm, and 4.80 N/mm, respectively.

### Model parameters: Influence of moving mass and spatial orientation

Having estimated the mass of the table, the combined mass of table and payload (*m*_*t*_ = *m*_*T*_ + *m*_*P*_), which will be referred to as the moving mass, can be used as the independent variable. Fig 3 already led to the conclusion that the damping coefficient *b*_*t*_ increases with increasing payload. In the detailed analysis presented now, *all* shaker parameters are examined for a possible dependence on the moving mass. Besides, it is investigated to what extent the spatial orientation of the shaker matters.

Fig 4b illustrates how the moving mass affects the resonance frequency (from now on measured in Hz rather than rad/s). Both axes are logarithmic, and since Eq (16) predicts a linear relationship between the logarithm of the moving mass and the logarithm of the resonance frequency, the data points should scatter around a straight line. This is indeed the case, as long as the moving mass does not exceed a certain limit. While this limit is around 100 g for the upright and the horizontal condition, the limit appears to be as low as 40 g for the upside-down condition. All in all, the horizontal condition shows the smallest deviation from linearity. The solid lines show the resonance frequencies that would be obtained if the parameter *k*_*t*_ were constant (the value of *k*_*t*_ was estimated from the data in Fig 4a).

A different view on the same data is obtained by converting the estimated resonance frequency into the spring constant *k*_*t*_ (using Eq (16)), which, in essence, characterizes the stiffness of the link between table and body. The results are displayed in Fig 4c. For comparison, the horizontal lines indicate the values that were estimated from the data in Fig 4a. The stiffness in the upright condition is, over the whole mass range, higher than the stiffness in the upside-down condition. When the moving mass increases above 200 g, the stiffness substantially increases in both cases. In the horizontal condition, by contrast, the stiffness is relatively constant.

**Fig 5:**
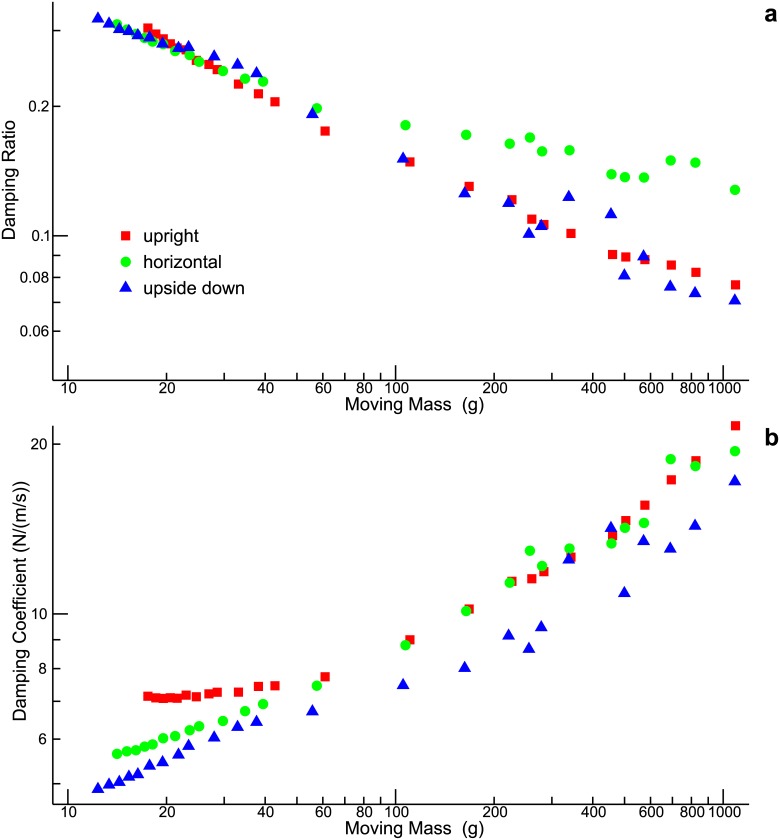
Parameter estimation (continued). Damping ratio **(a)** and damping coefficient **(b)** as functions of the moving mass.

Fig 5a shows the damping ratio, *ζ*_*t*_, as a function of the moving mass. As long as the moving mass is small (up to about 40 g), the damping ratio is roughly the same for the three spatial orientations. With increasing mass, however, the decrease of the damping ratio is less pronounced for the horizontal condition. Using Eq (24), the damping ratio can be converted into the damping coefficient, *b*_*t*_. The result is shown in Fig 5b. Unless the moving mass is small, the estimated damping coefficients are roughly the same for the upright and the horizontal condition. This is interesting in so far as the horizontal condition clearly differs from the other two conditions with respect to the damping *ratio*. The explanation can be found in Eq (24): The special position of the horizontal condition with respect to the damping ratio can be ascribed to the spring constant *k*_*t*_ (cf. Fig 4c).

**Fig 6:**
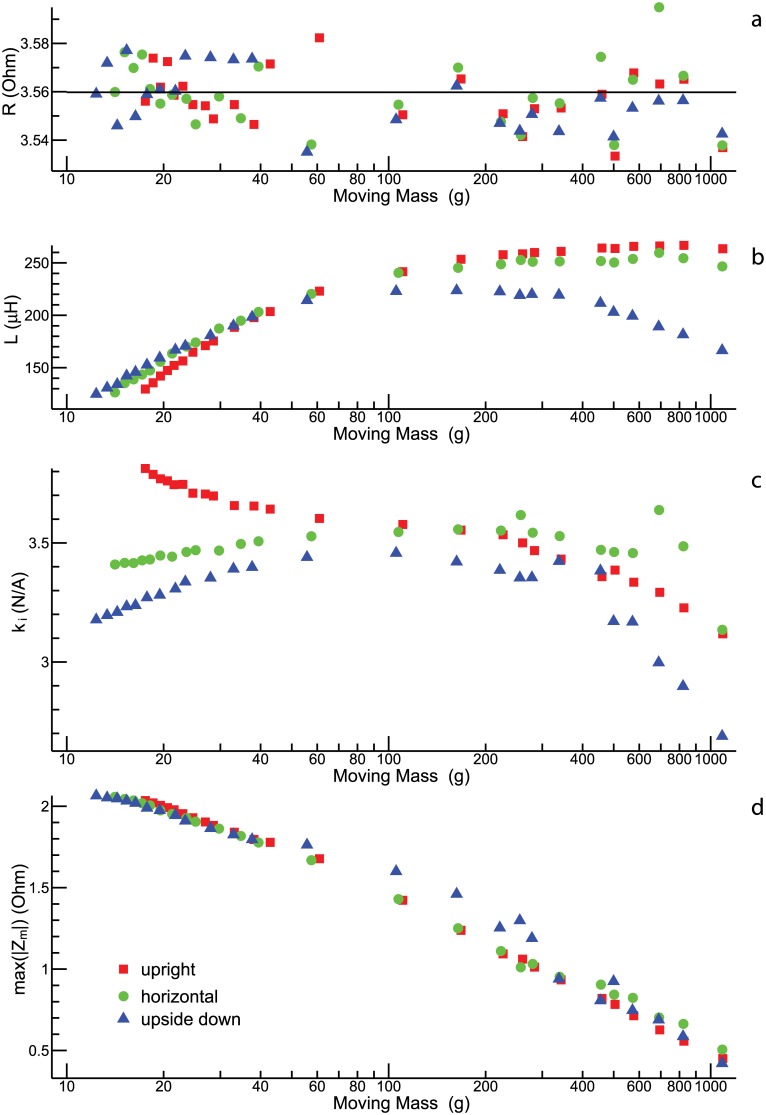
Parameter estimation (continued). Resistance *R* **(a)**, inductance *L* **(b)**, parameter *k*_*i*_ **(c)**, and max (|*Z*_*m*_|) **(d)** as functions of the moving mass.

The remaining model parameters are considered in Fig 6. The electrical resistance, *R*, shows no obvious dependence on the moving mass or the spatial orientation of the shaker (Fig 6a). The inductance *L* is difficult to determine from the given data, because the contribution of the term iωL to the overall electrical impedance is almost negligibly in the frequency range used for the fit. Thus, the estimates for *L* (Fig 6b) have to be interpreted with caution. Most reliable are the estimates obtained for a large moving mass: The larger the moving mass, the smaller is the resonance frequency, and the greater is the relative significance of the term iωL at frequencies much higher than the resonance frequency (for which the contribution of *Z*_*m*_ is small). Applying this thought to the results for the upright and the horizontal condition leads to the conclusion that *L* is about 250 *μ*H. The deviant results for the upside-down condition are unclear at this point.

By means of Eq (27), the estimates obtaned for ${\tilde{k}}_{i}^{2}$ can be converted into estimates for *k*_*i*_. The latter are shown in Fig 6c. All in all, the data suggest that *k*_*i*_ is about 3.5 N/A. The seeming dependence on the moving mass and the orientation of the shaker should not be overrated. First, the deviations from the mean appear more distinct than they really are (note the scale on the ordinate). Second, the estimation of *k*_*i*_ partially interferes with the estimation of other parameters. The latter argument is corroborated by Fig 6d, which shows max (|*Z*_*m*_|) as a function of the moving mass. The data displayed in that panel is calculated from the estimates for *k*_*i*_ and *b*_*t*_ (using Eq (31)). Part of the variability found in the plots for these two parameters (cf. Figs 5b and 6c) evidently cancelled out when calculating max (|*Z*_*m*_|).

### Level dependence

**Fig 7:**
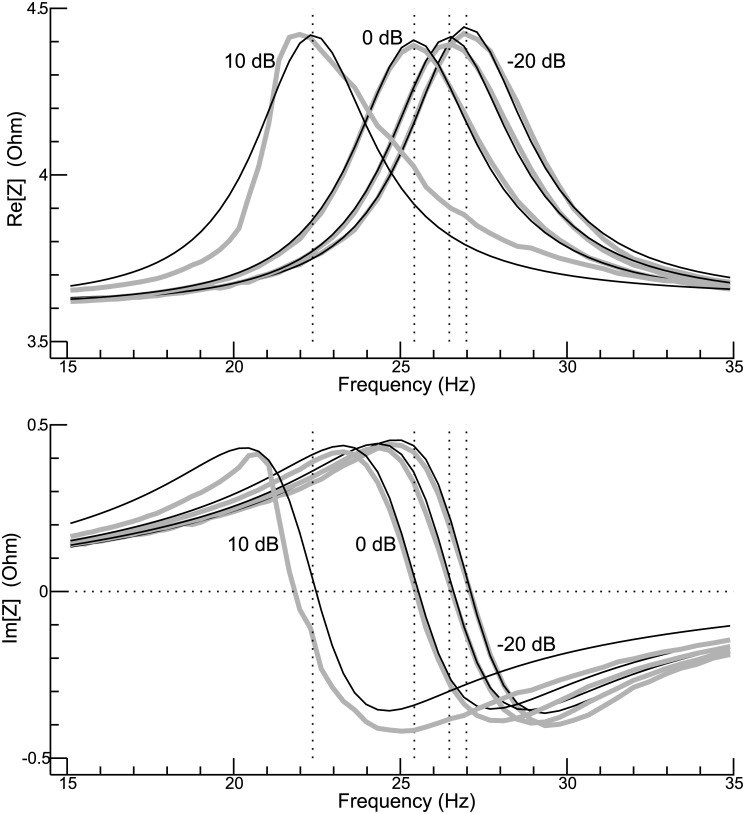
Level dependence of the electrical impedance. The real part of the impedance is considered in the upper panel, the imaginary part in the bottom panel. Grey curves represent the measured impedance, and black curves represent the model fit. The level was varied in steps of 10 dB between -20 dB and +10 dB, where 0 dB correspond to *E*_*i*_ = 1 V. The dotted vertical lines indicate the estimated resonance frequencies. The 500-g payload was used in this exemplary experiment.

If the shaker were a linear system, the electrical impedance would not depend on the input voltage. To see how well this applies, an exemplary experiment was done: The shaker was in an upright position, and the 500-g payload was used. The input voltage was varied, in steps of 10 dB, between -20 dB and +10 dB, where 0 dB (the default level in this study) corresponds to a maximum power-amplifier output of 1 V. The grey curves in Fig 7 represent the measured impedance (real and imaginary part displayed in separate panels), whereas the black curves represent the model fit. At the three lowest levels, data and model agree reasonably well. Moreover, the shapes of the curves are similar, as are the estimated resonance frequencies (26.98 Hz, 26.47 Hz, and 25.42 Hz, respectively). However, the situation at +10 dB is a different one. Most notably, model and data fundamentally differ. As a consequence, the estimated resonance frequency (22.37 Hz) considerably deviates from the frequencies at which the the real part of the measured impedance has a maximum or the imaginary part is zero. This leads to the conclusion that the performance of the shaker becomes increasingly nonlinear when the level approaches +10 dB.

### Hand-held shaker

**Fig 8:**
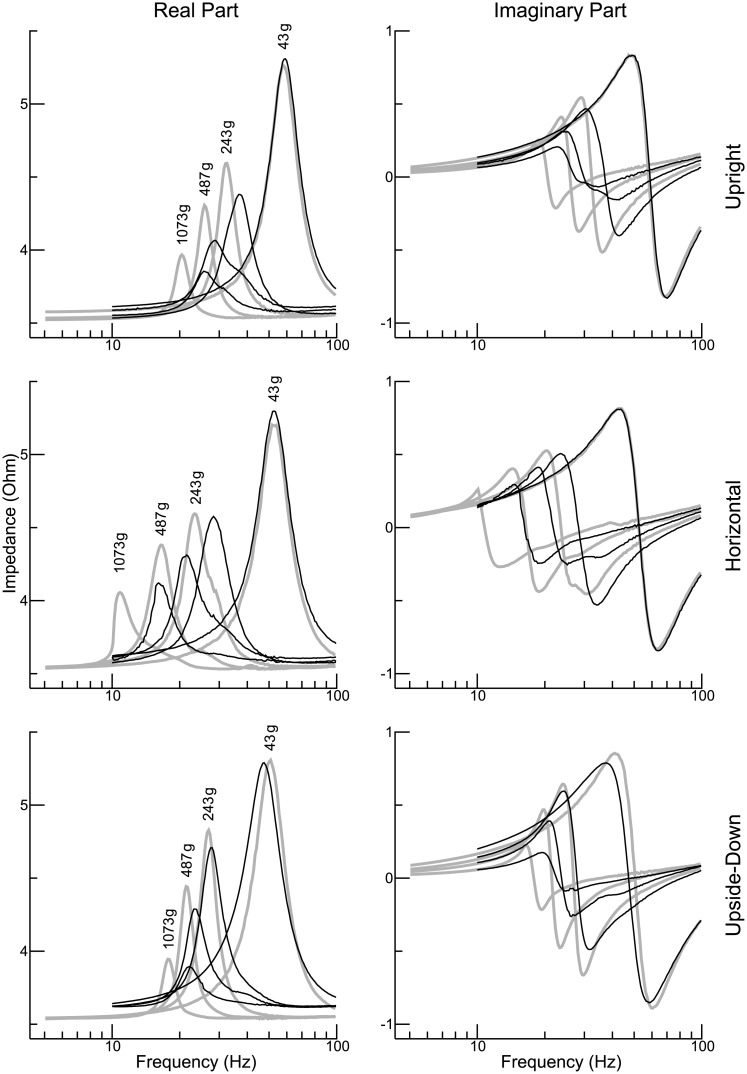
Electrical impedance of a hand-held shaker. Three different spatial orientations are considered: upright (top), horizontal (middle), and upside-down (bottom). The real part of the impedance is considered in the left column, the imaginary part in the right column. The black curves represent the hand-held shaker, whereas the grey curves show, for comparison, the corresponding results for the shaker firmly mounted on a solid foundation. Payloads between 43 g and 1073 g were investigated.

In the experiments analysed so far, the shaker was firmly mounted on a solid foundation. With respect to the application that motivated this study (measurement of VEMPs), the question arises as to what extent the dynamical properties of a *hand-held* shaker differ. The exemplary experiment considered in Fig 8 gives a first answer. The rows of the figure correspond to the three spatial orientations that were investigated. The real part of the impedance is shown on the left and the imaginary part on the right. The black curves represent the impedance of the hand-held shaker, whereas the gray curves represent the corresponding reference conditions (shaker mounted on the foundation). Four different payloads were investigated: 43 g, 243 g, 487 g, and 1073 g. The results for the upright and the horizontal condition agree in that the hand-held shaker and the mounted shaker have roughly the same resonance frequency in the case of the lowest payload, and that in the other three cases the resonance frequency of the hand-held shaker is significantly higher than that of the mounted shaker. The upside-down condition concurs as to the latter aspect, but for the lowest payload it is now the mounted shaker which shows the highest resonance frequency. A finding common to the upright and the upside-down condition is that the impedance curves of the hand-held shaker show, for the three highest payloads, peaks with reduced amplitudes.

### Exemplary accelerometer measurements

**Fig 9:**
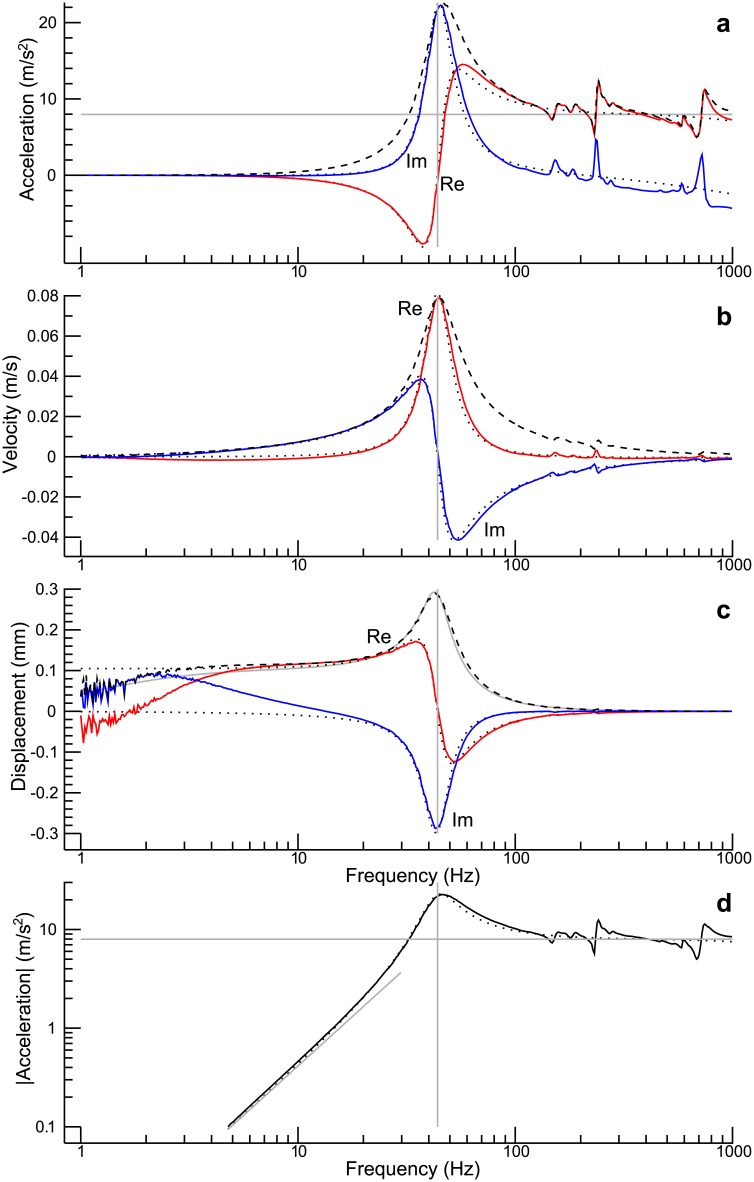
Comparison between model prediction and direct measurement with an accelerometer. The shaker was in an upright position, and the moving mass was 118 g. **(a)** The real and the imaginary part of the measured acceleration are represented by the solid curves, whereas the magnitude is represented by the dashed curve. The dotted curves show the model predictions for the real and the imaginary part. **(b)** Velocity calculated from curves in (a). **(c)** Displacement calculated from curves in (a). The additional grey curve shows an alternative model prediction of the magnitude of the displacement: The current actually measured rather than the predicted current was used in this calculation. **(d)** Magnitude of the acceleration plotted using logarithmic scales for both axes. The solid black curve represents the measured data, and the dotted curve shows the model prediction. The vertical grey line represents the resonance frequency estimated from the measured impedances (43.9 Hz). The other grey lines represent a low-frequency approximation (Eq (38)) and a high-frequency approximation (Eq (39)).

After having estimated the model parameters from the measured impedances, the model can be used to make predictions as to the displacement, the velocity, and the acceleration of the moving element of the shaker. These quantities can, of course, be measured also directly, and a comparison between prediction and direct measurement offers the opportunity to check the consistency of the model. An exemplary comparison is provided in Fig 9. An accelerometer was clamped into a miniature vice that was attached to the table of the shaker, and this construct then served as the payload (yielding a moving mass of about 118 g). The shaker was in an upright position. The solid curves in Fig 9a show the real part (“Re”) and the imaginary part (“Im”) of the measured acceleration as functions of frequency. The dotted curves represent the corresponding model predictions. The latter are based on Eq (14), with *I* calculated from Eq (13). Model prediction and data are in good agreement up to about 150 Hz. At higher frequencies, the measured acceleration shows several resonances, which are presumably due to vibrations of structural elements of the miniature vice. For the sake of completeness, the figure also shows the absolute value of the measured acceleration (dashed curve). The vertical grey line represents the resonance frequency estimated from the measured impedances (43.9 Hz).

Dividing the complex amplitude of the acceleration by iω and −*ω*^2^, respectively, yields the complex amplitudes of velocity and displacement. Fig 9b corresponds to Fig 9a, except that the velocity rather than the acceleration is considered. All in all, the curves derived from the measured accelerations are in good agreement with those derived from the model, particularly since the resonances at higher frequencies are not so conspicuous anymore. The corresponding curves for the displacement are shown in Fig 9c. For frequencies higher than about 30 Hz, the curves derived from the measured accelerations are again consistent with the corresponding model predictions. However, discrepancies can be noticed at low frequencies. A conspicuous, but unproblematic finding is that the displacement derived from the measured acceleration appears rather noisy at the lowest frequencies. This is caused by the fact that the calculation of the displacement involves a division by the squared frequency, which dramatically increases the noise level for the lowest frequencies. More serious is another discrepancy: While the model predicts a basically constant displacement at low frequencies, the displacement derived from the measured acceleration (see the dashed curve) shows a decreasing magnitude with decreasing frequency, particularly for frequencies below 3 Hz. But a significant deviation from the model prediction can already be seen around 10 Hz: The displacement derived from the measured acceleration shows an increasing imaginary part, whereas according to the model, the imaginary part should become zero in the low-frequency limit.

The explanation for these discrepancies is that the model does not account for the frequency characteristics of the power amplifier. Indeed, according to the manufacturer’s specifications, the lower 0.5-dB frequency of the amplifier is 10 Hz, and the lower 3-dB frequency is 4 Hz. To corroborate the argument that the observed discrepancies are caused by technical limitations of the power amplifier rather than a deficiency of the shaker model, the displacement *X*_*t*_ was calculated in an alternative way. The calculation was again based on Eq (14), but instead of calculating the current using the model, the current actually measured was used. The idea was that limitations of the power amplifier would be accounted for by this means. The results basically confirm this hypothesis: The magnitude of the predicted displacement (grey curve) is, indeed, roughly consistent with the magnitude of the displacement calculated from the measured accelerations (dashed curve). Considering the fact that the investigated frequencies are well within the operating range of the accelerometer (having a low-frequency limit of 0.3 Hz), it may be presumed that the remaining discrepancies are mainly caused by limitations of the current monitor built into the Brüel & Kjær power amplifier (type 2718): At low frequencies, the signal provided by this monitor may not accurately reflect the true current.

To better visualize the accelerations measured at low frequencies, Fig 9d shows the magnitude of the acceleration on a logarithmic scale. Moreover, grey lines represent the low-frequency approximation given in Eq (38) and the high-frequency approximation given in Eq (39), respectively. These approximations offer a simple way to estimate the constant *c* defined in Eq (33), provided that either *k*_*t*_ or *m*_*t*_ is known. In the present case, such estimations are, of course, not required anymore, because, using the parameter values estimated before, *c* can be determined directly from Eq (33). After rounding to one decimal place (*R* = 3.5 Ω, *k*_*i*_ = 3.5 N/A), we get *c* = 1 N/V. Thus, according to the high-frequency approximation given in Eq (39), an amplifier input of 10 V results in a force of 10 N. The latter value corresponds exactly to the the sine-peak force rating in the manufacturer’s specification.

### Static displacement of the table of the shaker

**Fig 10:**
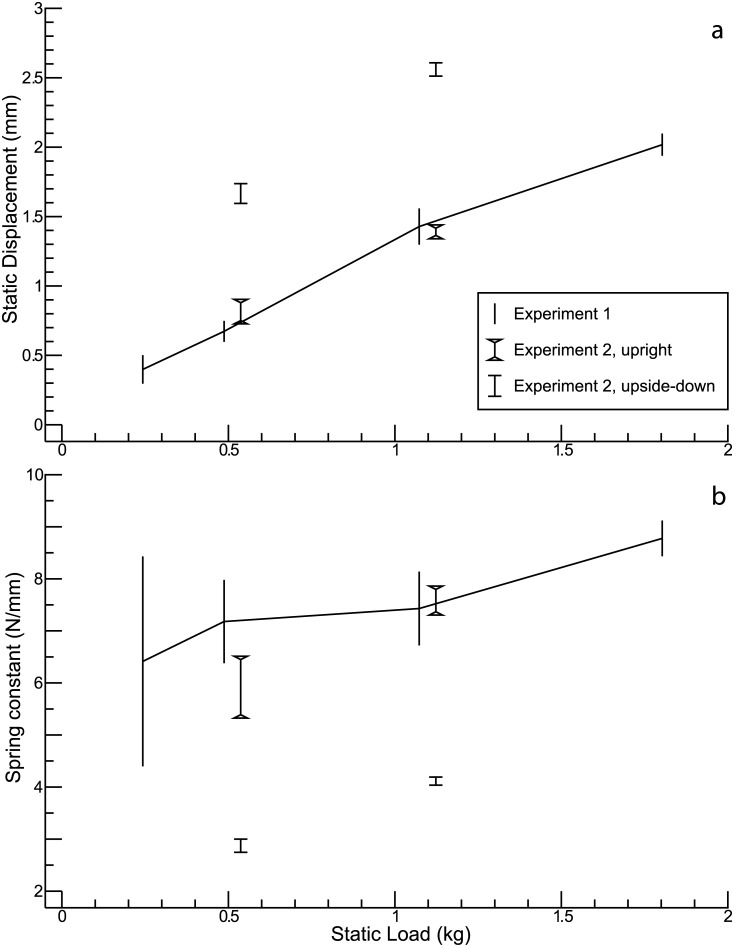
Measurements of static displacements. **(a)** Static displacement as a function of the static load. **(b)** Spring constants estimated from static displacements.

In a first experiment, which was done with the shaker in an upright position, the distance between the bottom of the shaker and the upper surface of the table was measured for different static loads. A measurement without load served as reference. The curve in Fig 10a shows the displacement of the table as a function of the load. Dividing the weight force by the resulting displacement yields an estimate of the spring constant, which is represented by the curve in Fig 10b. Under the static conditions investigated here, the spring constant shows, at most, a minor increase with increasing load.

In a second experiment, the distance between the bottom of the shaker and the upper surface of the static load was measured for the upright, the horizontal, and the upside-down condition. The horizontal condition then served as reference for calculating the displacement caused by the load. Two different loads (0.487 and 1.073 kg) were used in this experiment. The results of this second experiment are also visualized in Fig 10, although slightly shifted along the horizontal axis, to avoid overlapping with the results of the first experiment. The style of the error bars (indicating ± one standard deviation) allows for distinguishing the different conditions (see inset of Fig 10a). The results for the upright condition are roughly consistent with those of the first experiment. What is remarkable, however, is that the spring constants calculated for the upside-down condition are considerably smaller than those obtained for the upright condition.

## Discussion

### Model

The theoretical part of this article is based on the work of Doebelin [11], who investigated driven harmonic motion for a simple shaker model. He already provided a formula relating the table displacement *X*_*t*_ to the voltage *E*_*i*_ (see Eq 10.5 on page 417 of his book), but that formula is rather complicated, difficult to verify (the author himself characterized his algebraic reduction as “tedious and error-prone”), and inconvenient for further analytical considerations. Moreover, the solution is incomplete because formulas for the other two unknowns in the model (the current, *I*, and the displacement of the coil, *X*_*c*_) are missing. The model investigated here is a slightly extended version of the Doebelin model in so far as the body of the shaker is allowed to move relatively to the foundation (in the terminology of the present article, the original Doebelin model corresponds to the limit *k*_*b*_ → ∞, for which Eq (6) yields $1/{\bar{\rho }}_{tb}=0$). The experimental data analysed by Doebelin clearly show a resonance associated with the coupling between coil and table. With the parameter values he published, a resonance frequency of 3375 Hz is estimated using Eq (16). However, the Brüel & Kjær mini-shaker investigated in the present study does not have such a resonance in the frequency range investigated. The model could therefore be simplifed by assuming that coil and table are rigidly attached to one another.

### Shaker as a velocity sensor

In principle, the basic properties of a shaker could be easily determined by means of the “pluck” test suggested by Lang [12]: The cable which normally connects the shaker to the power amplifier is plugged into a device capable of monitoring the coil voltage, and using this setup the response to an abrupt release of the table of the shaker (held under pressure up to that time) is investigated. The induced voltage is proportional to the speed of motion, which means that the shaker can serve as a velocity sensor (cf. Eq (4) with *i* = 0 and *di*/*dt* = 0). By “plucking” the table of the shaker, a sine wave with an exponentially decaying envelope is elicited. Two “pluck” tests, one performed without a payload and another one with a known payload, are theoretically sufficient to characterize the mechanical properties of a simple shaker [12]. But because developing standardized experimental testing procedures deemed difficult, this approach was not pursued here. Instead, the electrical impedance was measured while the power amplifier drove the shaker at various frequencies. Although this procedure appears to have little in common with the “pluck” test, the two methods are related from a theoretical point of view: The velocity-dependent term in Eq (4), on which the “pluck” test is founded, is associated with *Z*_*m*_ in Eq (10), being of pivotal interest in the present study. Thus, *Z*_*m*_ can be understood as a signature of the “velocity sensor” inherent in the shaker.

### Parameter estimation from experimental data

The experimental investigations aimed to find out how the basic properties of the shaker are affected by external circumstances, particularly by the payload attached to the table and the spatial orientation of the shaker. Unless the stimulation level was too high (as was the case for the 10-dB condition in Fig 7), the model reasonably explained the data for each experimental condition. This proves the adequacy of the model and ensures that the estimated model parameters have a meaningful interpretation.

The dependence of the estimated parameters on external factors turned out to be more complicated than originally anticipated, and without knowing constructional details of the shaker it appears to be impossible to give comprehensive explanations for the observed effects. Generally speaking, one has to bear in mind that a real shaker is a much more sophisticated device than the simple model suggests. While there is only a single spring and a single damper in the model, the corresponding structures in the real shaker are spatially distributed and presumably consist of multiple components. Moreover, the assumption that the vibration is strictly confined to a single axis is certainly an idealization. If external conditions have some influence on how the structural elements of the real shaker are positioned relative to each other (which appears to be a realistic assumption), the way these elements interact is affected, and the resulting modifications of the overall properties of the shaker are finally seen in the model parameters estimated from the measured impedances.

After these general remarks, the individual model parameters are briefly discussed one after the other. The spring constant *k*_*t*_ (Fig 4c) does not depend on the moving mass as long as the latter is small. But, by all means, the spatial orientation of the shaker matters. A plausible explanation for the latter finding is that the suspension of the table of the shaker differs for different spatial orientations. Another finding is that *k*_*t*_ increases when the moving mass exceeds a certain limit, although only for the upright and the upside-down condition. This observation can probably be attributed to the fact that the equilibrium position of the table depends on the payload unless the shaker axis is horizontally oriented. The data can then be interpreted to the effect that the suspension of the table exhibits a higher stiffness once a heavy payload shifted the equilibrium position towards the largest possible displacement. These results differ from those derived from the static displacement (Fig 10b): A pronounced dependence on the static load is missing in the latter case. But the estimated spring constants have at least the same order of magnitude. Moreover, the dynamic and static measurements consistently led to the conclusion that the stiffness for the upright orientation is substantially greater than the stiffness for the upside-down orientation.

The damping coefficient *b*_*t*_ likewise depends on both the moving mass and the spatial orientation of the shaker (Fig 5b), although it is difficult to find a conclusive and unambiguous interpretation for the observed dependencies. A possible reason for this difficulty is that part of the observed damping could have origins that one would not presume in the first place. A conceivable damping mechanism is, for example, that the air within the shaker is forced to expand and compress as the table and the coil move, which would cause a loss of heat energy with each compression/expansion cycle [12]. Another mechanism could be friction. While the damping incorporated into the model is associated with a force that is proportional to the velocity of the moving object, friction is associated with a constant force. In classical friction experiments, this force is proportional to the weight of the moving object. Thus, the assumption of friction could help to explain why the estimated damping coefficient generally increases with increasing moving mass. Although it would be relatively easy to implement friction in the differential Eqs (1) to (3), such a modification of the model would enormously complicate the conisderation of driven harmonic motion. The much simpler case of an oscillator damped by a constant-magnitude friction force was considered by Marchewka et al. [27].

A noteworthy point is that the spatial orientation of the shaker has, for a small moving mass, almost no influence on the damping *ratio* (Fig 5a). Thus, from Eq (24) it can be concluded that the considerable influence on the damping *coefficient* is essentially due to the dependence of the spring constant *k*_*t*_ on the spatial orientation.

As one would expect, the electrical resistance, *R*, is basically independent of the moving mass and the spatial orientation of the shaker (Fig 6a). To what extend this also holds for the inductance *L* (Fig 6b) is, for methodological reasons that were explained when describing the results, difficult to decide. Methodological issues probably affected also the estimates obtained for the parameter *k*_*i*_. But this does not exclude the possibility that part of the observed variability reflects real effects: By influencing the position of the coil with respect to the permanent magnet, the spatial orientation of the shaker and the mass attached to its table perhaps affected the force developed by the shaker for a given current.

### Nonlinearity

Nonlinearity was not a central issue in this study. Nevertheless, an exemplary investigation of the level dependence of the electrical impedance revealed some nonlinear effects (Fig 7). Most conspicuous is the decrease of the resonance frequency with increasing level. At low levels the effect is small: The resonance frequencies at -20 dB and 0 dB differ by only about 1.5 Hz. But increasing the level from 0 dB to 10 dB lowers the resonance frequency by about 3 Hz. Probably more important is that the model cannot reasonably explain the 10-dB data. The presumed reason is that, during certain phases of the harmonic motion, the displacement of the table approached hard limits set by the design of the shaker: The maximum displacement according to the manufacturer’s specifications is 2 mm. [More precisely, the maximum peak-to-peak displacement is 4 mm. If the sign of the displacement did not matter, this specification could be interpreted as meaning that the maximum displacement from a suitably defined baseline (e.g, the equilibrium position when there is no payload) is ±2 mm. However, considering the fact that the estimated spring constant significantly differs for the upright and the upside-down condition, this interpretation of the peak-to-peak displacement may be too simple.] This displacement limit would have been exceeded also in the experiment considered in Fig 9c if the level had been 20 dB higher, because, in a linear approximation, such a level increase leads to a tenfold increase of the displacement. It should be noted, however, that the risk of overdriving the shaker is confined mainly to the vicinity of the resonance frequency.

Unless the shaker is horizontally oriented, a payload inevitably causes a static displacement. For the harmonic motion of the table this implies a shifted equilibrium position, which entails that the maximum possible displacement from the equilibrium position is reduced in the direction of the shift. From Fig 10a it can be concluded that in the case of the experiment considered in Fig 7 (shaker upright, payload of 0.487 kg) the equilibrium position was shifted by about 0.7 mm. As 1.3 mm displacement still remain for the driven motion, such a shift should be unproblematic in practice (unless the shaker is driven at frequencies near the resonance frequency). But much larger shifts of the equilibrium position are to be expected in typical VEMP experiments, in which the shaker is pressed against the test person’s head with a force of 10–20 N [2, 3]. For the upper limit of this range, Fig 10a predicts a shift of more than 2 mm so that pronounced nonlinear effects are to be expected for the driven motion of the table.

### Peculiarities of a hand-held shaker

The comparison between a hand-held shaker and a shaker firmly mounted on a solid foundation (Fig 8) revealed considerable differences. The example demonstrates, in particular, that the resonance frequency of the moving element of the shaker changes as soon as the body of the shaker is hold in the hand of an investigator. Quantitative modeling of the data obtained for the hand-held shaker is clearly beyond the scope of this study. But Eq (22) provides at least a qualitative explanation for the observation that the resonance frequency of a hand-held shaker is generally higher than that of a solidly mounted shaker (the results for the lowest payload represent an exception). For that purpose, the parameter *m*_*b*_ is reinterpreted as the combined mass of the body of the shaker and the hand of the investigator, whereas the spring constant *k*_*b*_ is used to characterize the coupling between the hand and the body of the investigator (the latter representing the “foundation” now). As to the latter parameter, the exact value does not matter as long as the table of the shaker has a resonance frequency (*ω*_*t*_) that is at least an order of magnitude higher than (*k*_*b*_/*m*_*b*_)^1/2^. Eq (22) correctly predicts that the relative discrepancy between the resonance frequencies of the hand-held shaker and the firmly mounted shaker increases with increasing mass *m*_*t*_.

## Conclusions and perspectives

Although small electrodynamic shaker have gained significant importance for VEMP investigations, their performance under typical measurement conditions, which greatly differ from the conditions referred to in the manufacturer’s specifications, is largely unknown. This study showed that the properties of an electrodynamic shaker can be determined without requiring special sensors such as accelerometers or force gauges: In essence, the shaker itself can serve as a vibration sensor, and the signature it leaves in the electrical impedance can be used to adjust the parameters of a simple model. The theory developed in this article is quite general. Thus, it is not only applicable to the special application that motivated the present study, but could be useful also in other contexts.

The theoretical framework was successfully applied to experimental data obtained for a Brüel & Kjær mini-shaker, which is the shaker generally used in VEMP studies. It turned out that the properties of this shaker substantially depend on the experimental conditions. As to the estimated model parameters, particularly the spring constant and the damping coefficient are affected. With respect to VEMP measurements it can be assumed that the performance of a shaker does not only dependent on its spatial orientation, but also on the force by which its moving element is pressed against the test person’s head. If this force is too large, the table of the shaker is statically displaced to such an extent that the maximum possible displacement is reached even when there is no electrical input. The shaker response to an electrical input can be expected to be fundamentally nonlinear under such circumstances. With that in mind, a force of 20 N, which represents the upper bound of the present recommendation for VEMP measurements [2, 3], could be a bit too high. The problem highlights, in any case, the desirability of a proper standardization of VEMP measurements.

While this study was almost exclusively concerned with driven harmonic motion, the shaker model is applicable to arbitrary electrical input signals, thus providing a means to design an electrical input so that the mechanical stimulus provided by the shaker is optimal in some sense. The idea to use a valid shaker model for tailoring the voltage *e*_*i*_ such that the displacement *x*_*t*_, the velocity ${\dot{x}}_{t}$ or the acceleration ${\ddot{x}}_{t}$ has a desired waveform has already been proposed by Doebelin [11]. Simulations of that kind would be based on Eqs (1) to (4). With respect to VEMP measurements it would, of course, be preferable if the reaction of the test person’s head to stimulation with the shaker could be simulated. Studies of bone conduction hearing [28, 29] showed that the head essentially behaves as a rigid body for low-frequency stimuli (e.g., a 200 Hz-tone), which means that the head basically moves as a whole without significant deformation. Higher frequencies, by contrast, give rise to quite complicated vibration patterns exhibiting numerous resonances and antiresonances [30–33]. While a detailed simulation of the latter situation requires a sophisticated model [34], whole-head movements can probably be described by relatively simple models. Thus, for a rough prediction of such movements it may be sufficient to supplement Eqs (1) to (4) by just one or two other differential equations.
